# A Paradigmatic Interplay between Human Cytomegalovirus and Host Immune System: Possible Involvement of Viral Antigen-Driven CD8+ T Cell Responses in Systemic Sclerosis

**DOI:** 10.3390/v10090508

**Published:** 2018-09-18

**Authors:** Maria-Cristina Arcangeletti, Clara Maccari, Rosanna Vescovini, Riccardo Volpi, Dilia Giuggioli, Gianluca Sighinolfi, Flora De Conto, Carlo Chezzi, Adriana Calderaro, Clodoveo Ferri

**Affiliations:** 1 Virology Unit, University-Hospital of Parma, Department of Medicine and Surgery, University of Parma, 43126 Parma, Italy; clara.maccari@studenti.unipr.it (C.M.); flora.deconto@unipr.it (F.D.C.); carlo.chezzi@unipr.it (C.C.); adriana.calderaro@unipr.it (A.C.); 2 Department of Medicine and Surgery, University of Parma, 43126 Parma, Italy; rosanna.vescovini@unipr.it (R.V.); riccardo.volpi@unipr.it (R.V.); 3 Rheumatology Unit, Medical School, University of Modena and Reggio Emilia, University-Hospital Policlinico of Modena, 41121 Modena, Italy; giuggioli.dilia@policlinico.mo.it (D.G.); gianluca.sighinolfi@gmail.com (G.S.); clodoveo.ferri@unimore.it (C.F.)

**Keywords:** human cytomegalovirus, virus-host interactions, systemic sclerosis, antigen-driven T cell responses, disease duration, modified Rodnan skin score

## Abstract

Human cytomegalovirus (HCMV) is a highly prevalent opportunistic agent in the world population, which persists as a latent virus after a primary infection. Besides the well-established role of this agent causing severe diseases in immunocompromised individuals, more recently, HCMV has been evoked as a possible factor contributing to the pathogenesis of autoimmune diseases such as systemic sclerosis (SSc). The interplay between HCMV and immune surveillance is supposed to become unbalanced in SSc patients with expanded anti-HCMV immune responses, which are likely involved in the exacerbation of inflammatory processes. In this study, blood samples from a cohort of SSc patients vs. healthy subjects were tested for anti-HCMV immune responses (IgM, IgG antibodies, and T cells to peptide pools spanning the most immunogenic HCMV proteins). Statistically significant increase of HCMV-specific CD8+ T cell responses in SSc patients vs. healthy subjects was observed. Moreover, significantly greater HCMV-specific CD8+ T cell responses were found in SSc patients with a longer disease duration and those with higher modified Rodnan skin scores. Given the known importance of T cells in the development of SSc and that this virus may contribute to chronic inflammatory diseases, these data support a relevant role of HCMV-specific CD8+ T cell responses in SSc pathogenesis.

## 1. Introduction

Systemic sclerosis (SSc) is an autoimmune disease characterized by immunological (both humoral and cellular) abnormalities, vasculopathy, and excessive extracellular matrix deposition, which leads to fibrosis of the skin and internal organs [1,2,3,4,5,6]. The disease appears to be the result of a multistep and multifactorial process including immune system alterations as well as genetic and environmental factors [1,2,3,4,6,7].

An increasing body of evidence highlights a critical role for T cell responses in the pathogenesis of systemic sclerosis (SSc), which contributes to fibrosis modulation and vascular damage [8,9,10,11,12]. Nevertheless, the type and function of specific T cell subpopulations has not yet been well characterized in relation to the different phases of the disease [12]. In particular, while several studies have focused on the CD4+ T cell subsets being involved in the inflammatory phase and contributing to the tissue fibrosis through the release of different proinflammatory and/or profibrotic cytokines [13,14,15,16,17,18,19,20,21], few studies have deepened the role of CD8+ T cells. Recent studies highlight a predominant role of CD8+ T cell infiltrates with a typical CD28− phenotype in the lesional skin of SSc patients already in the early stages of the disease [2,22]. Furthermore, it has been shown that an increased IL-13 production by peripheral blood CD8+ T cells homing to the skin of SSc patients correlates with the extent of skin fibrosis, which is a typical feature of this autoimmune disease [23,24]. In contrast, only a few CD8+ T cells were found in normal skin [22,23]. Other interesting observations rely on the fact that CD8+ T cells found in peripheral blood of patients with SSc present an antigen-driven oligoclonal expansion even though antigen specificity is not yet known [8,25,26,27].

Persistent/latent viral infections such as human cytomegalovirus (HCMV) infection have been evoked as possibly involved in the pathogenesis of SSc [8,9,11,28,29,30]. HCMV is able to infect human fibroblasts and endothelial cells, which are considered the “hallmark” cells of SSc [31,32]. Furthermore, the prevalence of antibodies anti-HCMV is significantly higher in SSc patients than in normal individuals [30,33,34,35]. It is also worthy to note that HCMV has been described to contribute to the expansion of specific subsets of CD8+ T cells (mostly against pp65 and IE1 viral proteins) with age [36,37,38,39,40,41].

The aim of this study was to investigate the involvement of HCMV-specific CD4+ and CD8+ T cell responses in the development of SSc. We show data demonstrating statistically significant higher amounts of peripheral blood CD8+ T cell subsets specific to HCMV antigens (mostly pp65 and IE1) in SSc patients compared to healthy subjects. Similarly, HCMV antigen-driven CD8+ T lymphocyte subsets were found to prevail in SSc patients with a longer disease duration and in those with a higher modified Rodnan skin score (mRSS).

## 2. Materials and Methods

### 2.1. Study Population

This observational prospective study included 20 unselected SSc patients (16 women and 4 men, ranging from 37 to 73 years, median age: 54 years) who were consecutively referred to the Rheumatology Unit, University-Hospital Policlinico of Modena, Italy. All patients fulfilled the 2013 ACR/EULAR criteria for SSc and were classified, according to the extent of skin involvement in limited and diffuse SSc [42].

Clinico-epidemiological and laboratory investigations including the modified Rodnan skin score (mRSS) to evaluate the extent of skin fibrosis [43,44] and the main visceral organ involvement were carried out, according to standardized methodologies [1,4]. The clinical and serological features based on the time of the present study were thoroughly reviewed from patients’ medical records. Patients with a clinical diagnosis of more than six years were classified as late disease-patients, which was previously described in References [2,12]. Eighteen age-matched and gender-matched healthy subjects were enrolled as controls. The study was conducted in accordance with the Declaration of Helsinki. The protocol was submitted to and approved by the local Institutional Review Board and “Area Vasta Emilia Nord” Ethical Committee (Project identification code: 2742016). All participants subscribed an informed consent to be enrolled in the study.

### 2.2. HCMV Serology

Peripheral blood samples obtained from recruited patients and healthy subjects were analyzed for HCMV antibodies. IgG and IgM anti-HCMV were detected using an automated system (Enzygnost Anti-CMV/IgG and IgM ELISA kit, BEP^®^ III System, Siemens Healthcare GmbH, Erlangen, Germany), according to the manufacturer’s instructions.

### 2.3. Detection of HCMV-Specific T Cell Responses

Peripheral Blood Mononucleated Cells (PBMCs) were obtained by Ficoll density gradient centrifugation (VWR International PBI, Milan, Italy) from peripheral blood samples with heparin. PBMCs were resuspended at 5 × 10^6^ cells/mL PBMCs in Roswell Park Memorial Institute 1640 medium (Euroclone, Milan, Italy) supplemented with 10% heat-inactivated fetal bovine serum and antibiotics (100 U/mL penicillin, 100 μg/mL streptomycin). For each tube, 200 μL of PBMCs suspension was incubated with peptide pools (PepMixes, JPT Peptide Technologies, Berlin, Germany) dissolved in dimethyl sulfoxide (Sigma-Aldrich, Milan, Italy), dimethyl sulfoxide alone as a negative control, or staphylococcal enteroxin B (Sigma-Aldrich) as a positive control, and monensin (Golgi Stop; BD Biosciences/BD Pharmingen, Milan, Italy) in a standard incubator (37 °C, humidified 5% CO_2_ atmosphere). Peptide pools (15-amino-acid length, 11-amino-acid overlapped between adjacent peptides) spanning the entire sequence of three HCMV proteins, which were tested: the PepMix pp65 contains 138 peptides spanning the 65-kDa lower matrix phosphoprotein, the PepMix IE1 contains 120 peptides spanning the 55-kDa immediate-early protein 1, and the PepMix UL94 contains 84 peptides spanning the capsid-binding protein UL94. After 2 hours, brefeldin A (Sigma-Aldrich) was added and the stimulation was performed for the remaining incubation time of 14 hours. Surface staining and intracellular cytokine detection were performed in accordance with standard protocols. The cells were treated for 10 minutes (min) with 20 mM ethylenediaminetetraacetic acid, washed, and then stained with saturating amounts of the following antibodies (all from BD Biosciences): phycoerythrin (PE)-conjugated anti-CD4, allophycocyanin (APC)-conjugated anti-CD8, and peridinin chlorophyll protein complex (PerCP)-conjugated ant-CD3 (pan T cell marker). After 30 min of incubation at 4 °C in the dark, PBMCs were incubated for 10 min at room temperature with 1 mL of fluorescence-activated cell sorter (FACS) lysing solution (BD Biosciences). Then, cells were washed and treated for 10 min with FACS permeabilizing solution 2 (BD Biosciences). Cells were washed and stained with fluorescein isothiocyanate (FITC)-conjugated anti-interferon (IFN)-gamma (BD Biosciences) for 30 min at 4 °C in the dark. After a final wash, the samples were acquired on a two-laser FACSCalibur cytometer (BD Biosciences) and at least 0.5 × 10^6^–0.7 × 10^6^ events/tube were analyzed using CELLQuest software. Files were first gated on lymphocytes, identified by a characteristic forward angle and side scatter profiles, and then CD4+ T cells and CD8+ T cells were selected on a CD3 vs. CD4 plot or alternatively on a CD3 vs. CD8 plot. Subsequently, IFN-gamma secreting CD4+ or CD8+ T cell responses were defined as the percentage of IFN-gamma+ events in the stimulated samples after background subtraction (percentage of IFN-gamma+ events in the corresponding negative control).

### 2.4. Statistical Analysis

Statistical analyses were performed using Prism 7.0 (GraphPad Software Inc., La Jolla, CA, USA). The D’Agostino & Pearson test was used to verify the normality of samples. Non parametric correlation (Spearman’s) was computed to examine the relationships between T cell responses vs. disease duration and vs. mRSS. Non parametric tests (Mann-Whitney) were used to compare groups. Data were expressed as median and interquartile. A probability value of *p* < 0.05 was considered significant.

## 3. Results

### 3.1. Study Population

The main characteristics of the SSc patients and healthy subjects included in the study are shown in Table 1. In particular, four patients were affected by diffuse and 16 were affected by limited cutaneous SSc. The mRSS widely ranged from 0 to 22 (median 8) with severity and extent of cutaneous sclerosis. Interstitial lung disease revealed by radiological, spirometric, and single breath diffusing capacity for carbon monoxide (DLCO Sb%) alterations was observed in 11 patients. Heart involvement and esophageal dysfunction were detected in eight and 14 patients, respectively. 

The presence of HCMV antibodies was tested in the study population concomitant with the analysis of T cell responses. Serological analyses revealed that 17/20 (85%) were seropositive (16/20 HCMV IgG positive and IgM negative while 1/20 only IgM positive). With regard to healthy subjects, 15/18 (83%) were HCMV IgG positive and IgM negative (Table 1). HCMV seronegative SSc patients and healthy controls were analyzed for T cell responses and, as expected, no HCMV-specific T cell responses were observed [45]. They were always excluded from comparative studies and statistical analyses.

### 3.2. HCMV-Specifc CD4+ and CD8+ T Cell Responses

First of all, peripheral blood T lymphocytes including total CD3+, CD4+, and CD8+ T cells were analyzed in SSc patients vs. healthy subjects and no statistical differences were found between the groups (Figure S1).

**Figure 1:**
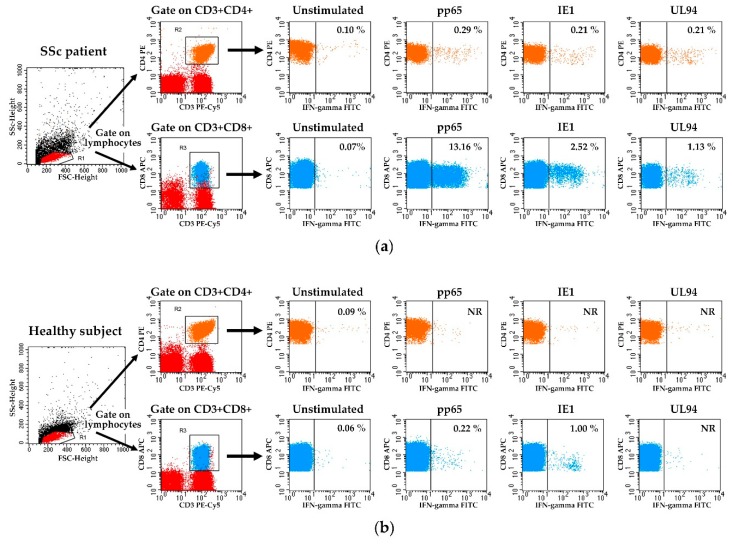
Representative plots of flow cytometry analysis displaying HCMV-specific CD4+ and CD8+ T cell responses in a SSc patient (**a**) and a healthy subject (**b**). PBMCs were stimulated for 16 hours with HCMV peptide pools and stained as described in the Materials and Methods section. Lymphocytes were identified by a characteristic forward angle (FSC-H) and side scatter (SSC-H) profiles. Then CD4+ and CD8+ T cells were gated on a CD3 vs. CD4 plot or alternatively on a CD3 vs. CD8 plot. Subsequently, IFN-gamma+ CD4+ or CD8+ T cell responses were defined as the percentage of IFN-gamma+ events in the samples stimulated with pp65, IE1, and UL94 peptide pools after subtraction of the percentage of IFN-gamma+ events in the corresponding unstimulated sample (NR = Non-Responder).

Then the analysis was focused on viral antigen-driven T cell responses. The presence of circulating CD4+ and CD8+ T cells specific to the most immunogenic HCMV antigens (i.e., the regulatory tegument phosphoprotein pp65 and the major immediate-early protein IE1) and also to the UL94 gene product (known to produce high antibody titres in SSc patients) was studied in SSc patients and healthy subjects. Figure 1 shows the gate strategy and representative plots of HCMV-specific T cells detection in a SSc patient (Figure 1a) vs. a healthy subject (Figure 1b). IFN-gamma+ CD4+ (orange) or CD8+ (blue) T cell responses were defined as the percentage of IFN-gamma+ events (shown in the upper right corner of each image) in the samples stimulated with HCMV pp65, IE1, and UL94 peptide pools after subtraction of the percentage of IFN-gamma+ events in the corresponding unstimulated sample. As can be observed (Figure 1a: SSc patient), CD8+ T cell responses were higher for pp65 and IE1 viral peptides than CD4+ T cell responses. On the other hand, no substantial differences in CD4+ and CD8+ T cell responses were observed for the healthy subject (Figure 1b).

**Figure 2:**
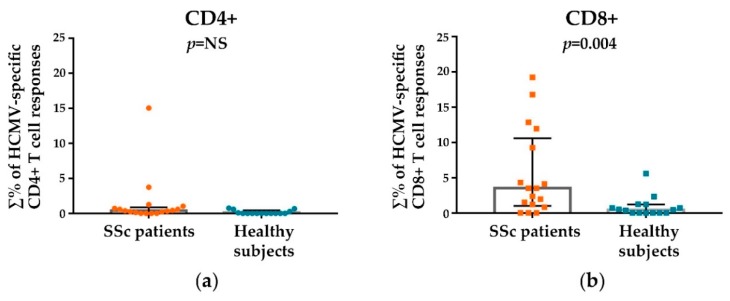
Total HCMV-specific CD4+ (**a**) and CD8+ (**b**) T cell responses in SSc patients compared to healthy subjects. HCMV-specific T-cell responses were examined by measuring intracellular expression of IFN-gamma after stimulation with pp65, IE1, and UL94. The percentages (∑%) reported were obtained by accumulating the individual percentage response to each stimulating peptide pool (pp65, IE1, UL94). For each scatter plot, median (column), and interquartile range are shown. The Mann-Whitney nonparametric test was used to derive *p* values (NS = not significant).

Figure 2 shows the analysis of the results of total HCMV-specific CD4+ and CD8+ T cell responses in SSc patients compared to healthy subjects. Total HCMV-specific CD4+ T cell responses (Figure 2a) were comparable in patients vs. healthy subjects (medians: 0.38% and 0.00% of total CD4+ T cells respectively, *p* = not significant). HCMV-specific CD8+ T cells (Figure 2b) showed a different response pattern: total HCMV-specific CD8+ T cell responses were significantly increased in SSc patients in comparison to those observed in healthy subjects (medians: 3.51% and 0.45% of total CD8+ T cells respectively, *p* = 0.004). 

Percentages of HCMV-specific CD4+ and CD8+ T cell responses in SSc patients and healthy subjects to total (∑%) and each (%) viral peptide are shown in Table 2. Within SSc patients, overall HCMV CD8+ T cell responses were higher than CD4+ T cell responses. HCMV CD8+ T cells were mostly directed to pp65 (4/12, patients 2, 6, 11, and 14) to IE1 (6/12, patients 3, 7, 8, 10, 13, and 17) or both (2/12, patients 9 and 15). The total percentages of these HCMV-specific CD8+ T cell responses were distributed in a quite large range spanning from 0.83% to 19.23% with a prevalence of high (19.23%, 16.81%, 9.27%, 11.95%) and medium (4.33%, 4.12%, 3.51%) values.

**Figure 3:**
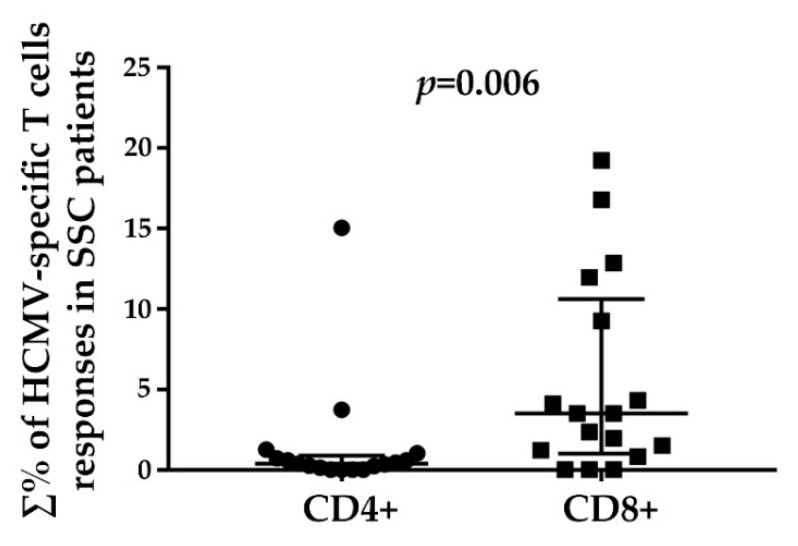
Relationship between total HCMV-specific CD4+ and CD8+ T cell responses in SSc patients. The percentages (∑%) reported were obtained by adding the individual percentage response to each stimulating peptide pool (pp65, IE1, and UL94). For each scatter plot, median and interquartile ranges are shown. The Mann-Whitney nonparametric test was used to derive *p* values.

The analysis of the overall percentage distribution of HCMV-specific CD4+ and CD8+ responses in SSc patients (Figure 3) revealed a statistically significant (*p* = 0.006) predominance of CD8+ T cell responses.

### 3.3. HCMV-Specific CD8+ T Cell Responses in Relation to the Disease Duration and Modified Rodnan Skin Score

**Figure 4:**
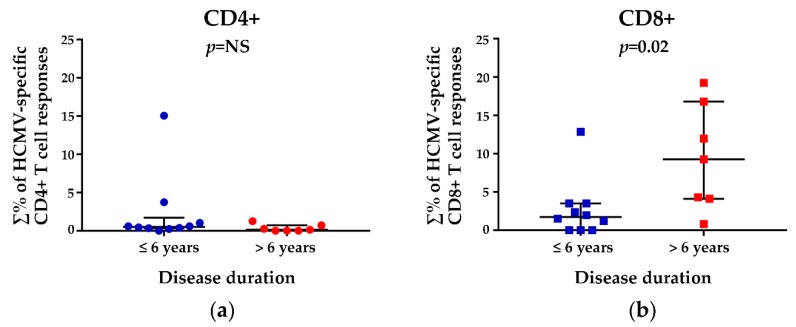
Relationship between total HCMV-specific CD4+ (**a**) or CD8+ (**b**) T cell responses and disease duration (≤6 years and >6 years). The percentages (∑%) reported were obtained by adding the individual percentage response to each stimulating peptide pool (pp65, IE1, and UL94). For each scatter plot, median and interquartile ranges are shown. The Mann-Whitney nonparametric test was used to derive *p* values (NS = not significant).

HCMV-specific T cell responses detected in SSc patients were first analyzed in relation to the disease duration (Figure 4). The results revealed that, while no significant differences in both groups (≤6 years and >6 years) were found for HCMV-specific CD4+ T cell responses (Figure 4a), SSc patients with a longer disease duration (>6 years) had a significantly increased HCMV-specific CD8+ T cell responses vs. patients with a disease duration ≤6 years (medians: 9.27% and 1.73% of total CD8+ T cells respectively, *p* = 0.02) (Figure 4b).

**Figure 5:**
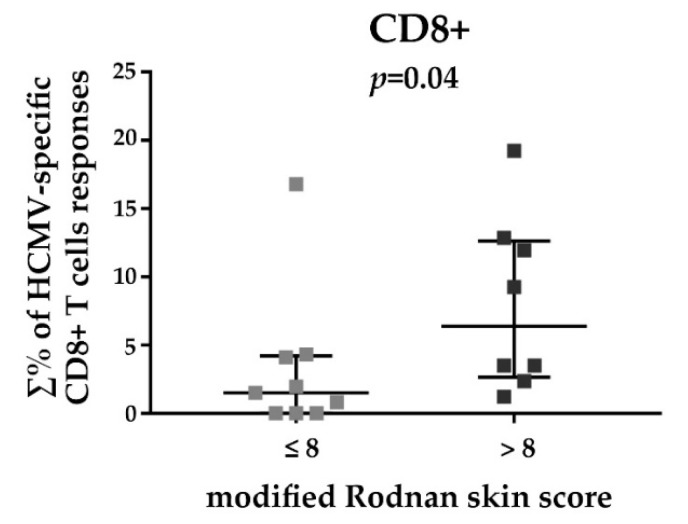
Relationship between total HCMV-specific CD8+ T cell responses and the modified Rodnan skin score (≤8 and >8). The percentages (∑%) reported were obtained by adding the individual percentage response to each stimulating peptide pool (pp65, IE1, and UL94). For each scatter plot, median and interquartile ranges are shown. The Mann-Whitney nonparametric test was used to derive *p* values.

HCMV-specific T cell responses detected in SSc patients were also analyzed in relation to a mRSS ≤8 or >8 (8 being the median value, see Table 1). The results are shown in Figure 5. SSc patients with a mRSS higher than 8 showed a significantly increased HCMV-specific CD8+ T cell responses compared to those with a mRSS ≤8 (medians: 9.27% and 1.73% of total CD8+ T cells respectively, *p* = 0.04). In this case, no differences between the considered mRSS sub-groups were found for HCMV-specific CD4+ T cell responses [45]. The mRSS values of the SSc study population were not correlated to the disease duration [45].

## 4. Discussion

Viruses that persist in the infected subject after primary infection such as HCMV, Epstein-Barr virus (EBV), and parvovirus B19 [28,29] have been proposed as possible triggering or worsening factors of SSc.

In particular, HCMV has been evoked as a possible contributing factor to the etiopathogenesis of SSc through its ability to infect the cells mainly involved in the disease, i.e., fibroblasts and endothelial cells [7,9,11,30,31]. For instance, the presence of HCMV RNA was demonstrated in a skin biopsy of a woman with SSc diagnosed after an acute HCMV infection [46]. Higher prevalence of anti-HCMV antibodies in SSc patients than in healthy subjects also constitutes indirect evidence for such an association [30,33,34,35]. Furthermore, a molecular mimicry mechanism by which antibodies against HCMV-derived UL94 protein can be likened to endothelial cell damage in SSc patients has been described [33,47].

It is also of note to remind that HCMV has a deep impact on the host immune system through its capacity to modulate host responses during its life cycle. Its persistence as a latent virus does not correspond to a silent condition but rather is a source of continuous stimulation for the immune system through rounds of subclinical reactivations mostly concerning virus-specific subsets of CD8+ T lymphocytes [48,49]. In particular, it has been observed that viral CD8+ T lymphocytes with an effector-memory phenotype increase in the number along latency. This phenomenon, which is a hallmark of CD8+ T cell responses to CMV (murine and human) infection, is known as “memory inflation”. On the contrary, CD4+ T cells do not seem to undergo consistent inflation [37,38,41]. This continuous commitment of immune effectors for controlling this agent exerts a heavy charge on the host. To this regard, several studies have indicated HCMV as involved in the development of chronic inflammatory diseases and detrimental effects on immunosenescence and health in elderly subjects [7,36,39,40,50].

Concerning “memory inflation”, it is also important to consider that not all members of the *Herpesviridae* family develop a phenomenon comparable to that elicited by HCMV despite their common feature to persist as latent viruses and reactivate in an infected subject [41,51]. With regard to EBV, which is another possible candidate that triggers several autoimmune diseases, the size of specific CD8+ T cells seems to be stable over time in the blood of healthy individuals and patients who had infectious mononucleosis and no sign of inflation compared to that occurring upon HCMV infection [51]. Even in the elderly, it has been reported that HCMV can drive much greater expansions of antigen-specific CD8+ T lymphocyte responses when compared with EBV [52]. Based on the above mentioned notions, it appears unlikely that viral antigen-specific CD8+ T cell inflation could occur upon EBV infection even though a role of the latter agent in SSc by the activation of other mechanisms has been described [53,54].

Considering what stated above, the aim of this study was to better define the involvement of HCMV in SSc by focusing on the viral-specific CD4+ and CD8+ T cell responses to the main immunogenic viral antigens pp65 and IE1. The product of the viral UL94 gene was also included.

CD8+ T cell responses have been described to be involved in the early stages of the disease by Fuschiotti and collaborators [23]. The authors observed IL-13-producing CD8+ T cells in mononuclear infiltrates in the skin of SSc patients and also a considerable number of CD8+ T cells expressing skin-homing receptors in blood, which suggests that CD8+ T cells have an important role in the initiation of the disease even though they may also contribute to disease progression since they remain present to a lesser extent in late SSc stages where CD4+ T cells seem to prevail [23]. In a more recent work, Fuschiotti also observed that CD8+ T lymphocytes in peripheral blood of SSc patients presented an antigen-driven oligoclonal expansion even though antigen specificity is not yet known. They were numerous in patients with active SSc, and likely contribute to the pathogenesis of the disease [12].

Accordingly, our data highlight the presence of HCMV antigen-driven CD8+ T cells (in particular, responsive to pp65 and IE1) in the blood of SSc patients where they were significantly more represented than in healthy controls. CD8+ T cell responses to the considered immunodominant viral antigens were also significantly higher in SSc patients when compared to CD4+ T lymphocytes. Coherent with our findings, Cicin-Sain and collaborators [55] observed that memory inflation in the murine cytomegalovirus model is an expansion of effector CD8+ T cells against few viral immunodominant epitopes (pp65 and IE1) at the expense of subdominant ones rather than a general increase of T cell responses to cytomegalovirus.

By contrast, a recent work carried out on Systemic lupus erythematosus and SSc patients by Janahi and collaborators [56] provides data supporting higher prevalence of HCMV-specific CD4+ than CD8+ T cell responses. The mean duration of SSc in the considered study population was less than six years (39 ± 18 months), which accounts for early stages of the disease. These data are apparently conflicting when compared to those obtained by Fuschiotti [12] and to ours.

In the case of Fuschiotti’s work, it has to be noted that expanded CD8+ T cell subsets observed in the early phases of SSc were described as antigen-driven even though the antigen specificity was not investigated.

Concerning our data, the significantly higher HCMV-specific CD8+ T cell responses in SSc patients vs. healthy subjects and vs. CD4+ T cell responses were also connected to the disease duration. In other words, while Janahi and collaborators found a prevalent presence of HCMV-specific CD4+ T cells in the early phases of SSc, the prevalence of viral antigen-driven CD8+ T lymphocytes arising from our work is significantly associated to a longer disease duration. Furthermore, the applied experimental protocols differed in that Janahi and collaborators used a pool of HCMV antigens consisting in a whole lysate derived from MRC5 fibroblasts infected with HCMV and then “purified” by centrifugation. One can assume that this suspension was enriched with viral progeny, but the presence of residual, soluble cellular components cannot be excluded. Most importantly, it has been demonstrated that, when whole proteins or viral lysates are used as stimulating antigens, CD4+ T cell responses are optimal while the process is generally inefficient for CD8+ T cell stimulation [57]. Maecker et al. also showed that the use of overlapping peptide mixtures as antigens including 15 amino acid peptides with 11 amino acid overlaps (as those used in our study) may be very useful for a balanced detection of both CD4+ and CD8+ T cell responses. By using overlapping peptide mixtures, we focused on immunodominant viral peptides including pp65 and IE1. Our choice is in accordance with the observations made on murine and human CMV where memory inflation has been found to be a response of CD8+ T cells directed to specific, immunodominant viral epitopes [55,58].

Our results are also coherent with HCMV serological data of the study population. Among 17 HCMV-seropositive SSc patients, 16 were IgG-positive/IgM-negative while 1 was only IgM-positive. This could mean that, for the large majority of the considered patients, the serological *status* is consistent with a non-recent HCMV infection and, likely, with a number of subclinical viral reactivations supporting increased HCMV antigen-driven CD8+ T cell populations comparing them to HCMV-positive healthy subjects [59].

The postulated higher frequency of HCMV reactivation rounds in SSc patients can be connected to the possible pathogenetic role of this viral agent by considering that: (i) the endothelial damage is one of the characteristic features of this disease, (ii) as already mentioned, HCMV is able to infect, among others, endothelial cells [31], and (iii) upon infection, endothelial cells undergo important changes and increased expression of adhesion molecules [60]. (iiii) It is also well established that peripheral blood monocytes represent one of the major HCMV latency sites and that their differentiation into macrophages can lead to viral reactivation [61,62]. It is most likely that monocytes harboring the latent virus might be attracted to and induced to trans-endothelial migration and monocyte-to-macrophage differentiation by an increased expression of adhesion molecules in HCMV-infected endothelial cells, which triggers virus reactivation and damage of neighboring and distal vascular tissues contributing to the seriousness of vascular injury in SSc [60,63]. In this context, increased runs of HCMV reactivation that are supposed to occur in SSc patients could explain the reinforced inflation of virus-specific CD8+ T cells and also prime them to exert their cytolytic properties on non-infected bystander cells as previously observed [64]. This worsens inflammatory processes.

Thus, the postulated pathogenetic role of HCMV in SSc could be exerted both by the direct infection of the hallmark cells of this autoimmune disease (in particular, but not only, endothelial cells) and by increased reactivation rounds leading to a reinforced virus specific CD8+ T cell inflation.

Another interesting observation from our study concerns the statistically significant connection between increased HCMV-specific CD8+ T cell responses and the highest mRSS of the study population. As known, the measurement of skin thickness by mRSS is used as a surrogate for disease activity and severity in patients with diffuse cutaneous SSc: mRSS worsening is associated with poorer patient outcomes [65,66,67,68].

Concerning the pharmacological treatment, the large majority of therapeutic agents administered to the considered SSc patients does not have any known direct effect on the immune system, which does not influence the results of our study. Only two patients got steroids, one of which was HCMV-seronegative (thus, excluded from the comparative analyses). The other one was treated with a very low dosage of 6-methyl-prednisolone.

Our data reinforce the hypothesis of HCMV involvement in the pathogenesis of this multifactorial autoimmune disease with the possible contribution of other infectious agents, environmental agents, and/or genetic co-factors. Their variable combination might be responsible for specific clinical phenotypes [1,2,3,4,28].

Considering the potential impact of the present findings, enhanced characterization of HCMV-driven CD8+ T cell responses and their relationships with clinical features in a larger cohort of SSc patients will lead to a deeper understanding of the pathogenesis of this autoimmune disease and also to better patient management and new therapeutic approaches.

## Figures and Tables

**Table 1:** Main characteristics of the study population.

	SSc Patients	Healthy Subjects
Number (n)	20	18
Age, years		
Median (range)	54 (37–73)	55 (39–62)
Gender, n (%)		
Male	4 (20)	2 (11)
Female	16 (80)	16 (89)
HCMV serology, n (%)		
IgG+ IgM-	16 (80)	15 (83)
IgG- IgM-	3 (15)	3 (17)
IgG- IgM+	1 (5)	
Disease duration, years		
Median (range)	4 (1–19)	
Clinical subgroups, n (%)		
Diffuse cutaneous	4 (20)	
Limited cutaneous	16 (80)	
Clinical manifestations, n (%)		
Raynaud’s phenomenon	20 (100)	
Digital ulcer	7 (35)	
Puffy fingers	10 (50)	
Pitting	7 (35)	
Telangiectasia	12 (60)	
Arthralgia	11 (55)	
Interstitial lung disease	11 (55)	
Heart involvement	8 (40)	
Esophageal dysfunction	14 (70)	
FVC% ^1^, median (range)	101 (86–130)	
FEV1% ^2^, median (range)	103 (84–126)	
DLCO Sb% ^3^, median (range)	65 (31–94)	
Modified Rodnan skin score		
Median (range)	8 (0–22)	
Autoantibodies, n (%)		
Positive ANA ^4^	19 (95)	
Positive ACA ^5^	8 (40)	
Positive anti-SCl70	5 (25)	
Treatment, n (%)		
Steroids	2 (10)	
Prostanoids	16 (80)	
Bosentan	5 (25)	
Calcium antagonist	12 (60)	

^1^ FVC = forced vital capacity, ^2^ FEV1 = forced expiratory volume in one second, ^3^ DLCO Sb = single breath diffusing capacity for carbon monoxide, ^4^ ANA = anti-nuclear autoantibodies, and ^5^ ACA = anti-centromere autoantibodies.

**Table 2:** HCMV-specific CD4+ and CD8+ responses in HCMV-seropositive SSc patients and healthy subjects.

Blood Samples	CD4+ T Cell Responses (%)				CD8+ T Cell Responses (%)			
	∑% ^1^	pp65	IE1	UL94	∑%	pp65	IE1	UL94
SSc pz ^2^ 1	15.06	3.11	5.28	6.67	12.86	2.65	5.20	5.01
SSc pz 2	0.25	0.25	NR ^3^	NR	19.23	19.23	NR	NR
SSc pz 3	0.59	NR	NR	0.59	1.21	NR	1.21	NR
SSc pz 4	3.74	0.52	NR	3.22	3.52	0.74	NR	2.78
SSc pz 5	1.04	0.43	0.45	0.6	NR	NR	NR	NR
SSc pz 6	0.71	0.29	0.21	0.21	16.81	13.16	2.52	1.13
SSc pz 7	1.26	0.4	0.28	0.58	9.27	4.08	4.64	0.55
SSc pz 8	NR	NR	NR	NR	4.33	0.98	3.35	NR
SSc pz 9	0.38	NR	0.1	0.28	1.96	1.1	0.86	NR
SSc pz 10	NR	NR	NR	NR	11.95	1.05	10.9	NR
SSc pz 11	0.13	0.13	NR	NR	4.12	3.13	0.77	NR
SSc pz 12	NR	NR	NR	NR	NR	NR	NR	NR
SSc pz 13	0.44	0.44	NR	NR	2.37	0.42	1.95	NR
SSc pz 14	0.23	0.23	NR	NR	3.51	3.36	0.15	NR
SSc pz 15	0.35	NR	0.13	0.22	1.51	0.61	0.90	NR
SSc pz 16	0.6	0.28	0.32	NR	NR	NR	NR	NR
SSc pz 17	NR	NR	NR	NR	0.83	NR	0.19	0.64
Hs ^4^ 1	NR	NR	NR	NR	NR	NR	NR	NR
Hs 2	NR	NR	NR	NR	NR	NR	NR	NR
Hs 3	0.67	0.33	0.23	0.11	1.23	0.5	0.45	0.28
Hs 4	NR	NR	NR	NR	NR	NR	NR	NR
Hs 5	0.60	NR	NR	0.60	0.71	0.71	NR	NR
Hs 6	NR	NR	NR	NR	5.60	5.60	NR	NR
Hs 7	0.77	0.56	0.08	0.13	0.72	0.72	NR	NR
Hs 8	NR	NR	NR	NR	NR	NR	NR	NR
Hs 9	NR	NR	NR	NR	NR	NR	NR	NR
Hs 10	NR	NR	NR	NR	1.22	0.22	1.00	NR
Hs 11	0.28	0.28	NR	NR	0.52	0.52	NR	NR
Hs 12	NR	NR	NR	NR	NR	NR	NR	NR
Hs 13	0.09	0.09	NR	NR	2.32	NR	2.32	NR
Hs 14	NR	NR	NR	NR	0.45	0.45	NR	NR
Hs 15	NR	NR	NR	NR	0.37	0.11	0.26	NR

^1^ ∑% = total HCMV-specific T cell responses obtained by adding the individual percentage response to each stimulating peptide pool (pp65, IE1, UL94), ^2^ pz = patient, ^3^ NR = Non-Responder, ^4^ Hs = Healthy subject.

## Supplementary Materials

The following are available online at http://www.mdpi.com/1999-4915/10/9/508/s1.
