# Supplementary material for: A Paradigmatic Interplay between Human Cytomegalovirus and Host Immune System: Possible Involvement of Viral Antigen-Driven CD8+ T Cell Responses in Systemic Sclerosis
Source: Viruses. 2018 Sep 18;10(9):508. doi: 10.3390/v10090508 (PMC6163388; doi:10.3390/v10090508)
Supplement: Supplementary file 1 [file viruses-10-00508-s001.pdf]

Supplementary Materials:

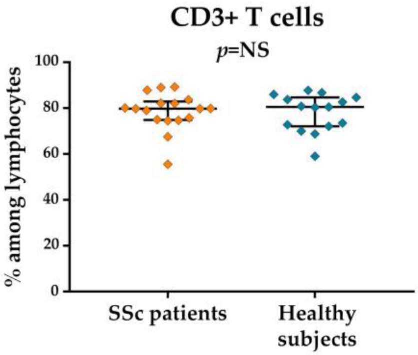

(**a**)

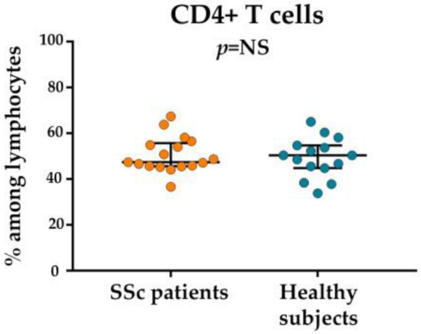

(**b**)

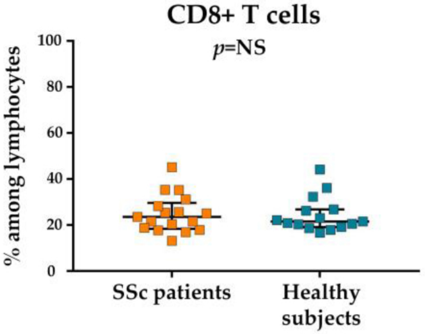

(**c**)

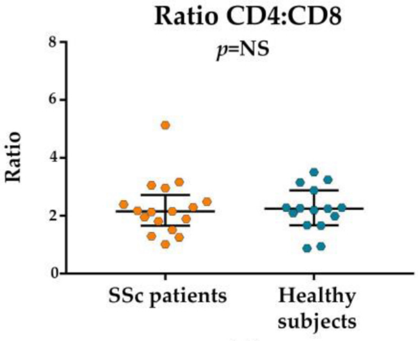

(**d**)

Figure S1. Analysis of lymphocyte subsets in SSc patients compared to healthy subjects: total CD3+ (**a**), CD4+ (**b**), CD8+ (**c**) T cells and ratio CD4:CD8 (**d**). For each scatter plot, median and interquartile range are shown. The Mann-Whitney nonparametric test was used to derive p values (NS=not significant).
